# A Carlsbad Physician on the Treatment of Gout

**Published:** 1904-05-21

**Authors:** 


### A Carlsbad Physician on the Treatment of Gout.

The clinical problem of gout appears to possess features which secure for it an ever enduring interest. Many of the most acute minds in the profession, both in ancient and modern times, have bent their best energies to its solution, and though new observations and experiments have been multiplied, there yet remains some factor which eludes definition. Even the more exact chemical knowledge of the present day has not succeeded in penetrating the heart of the mystery, and, indeed, it has, in some quarters, added fuel to the controversies which through long ages have burned in fierce flame round this strange malady. But a few months ago one of our monthly contemporaries devoted two successive issues to the publication of essays on the various aspects of the subject, and succeeded in illustrating the truth that amongst the greatest living authorities there still exists much difference both of opinion and practice. Possibly part of the attraction exercised by the question is to be found in the mystery which lies at the heart of it, and even though rival interpreters proclaim in somewhat over-confident tones the value of their several views, it is all to the good that by a free and full discussion the various theories and the facts which bear upon them should be placed in an authoritative fashion before the profession. One of the essays above referred to has a special title to respect inasmuch as it is from the pen of a physician who for more than thirty years has enjoyed the peculiar opportunities offered for the study of gout by practice at Carlsbad. Dr. London, as is evident from his paper, has accumulated a vast store of experience, and fortunately possesses both the art of utilising this for practical ends, and the capacity to summarise and present his knowledge in concise and luminous phrases. His freedom from dogmatism and the careful manner in which he qualifies his generalisations render all the more impressive his practical teaching. Turning to the substance of Dr. London's essay we note that he is content to present the various hypotheses which have been advanced in reference to the nature of gout without endeavouring to add to their number, though the brief comments he attaches to several of these are in every sense suggestive. Into the theoretical aspect of the much-disputed relationship between the various forms of chronic arthritis Dr. London does not enter, but he has no doubt that long-standing rheumatism of joints predisposes these articulations to subsequent attacks of gout and that the two diseases may produce mixed forms which are accurately termed "rheumatic gout" or "gouty rheumatism." Arthritis deformans, or as it is more generally termed in this country rheumatoid arthritis, he regards as having no etiological relationship to true gout, and he insists on the clinical distinctions which are generally allowed by British physicians. Heberden's nodes, on the contrary, he places without question among gouty phenomena though he does this on the authority of other physicians rather than on that of his own observations. Still his subscription to this view is obviously of importance more especially when his special experience is borne in mind. Heberden himself, it will be remembered, adopted a very impartial attitude towards the "digitorum nodi," which he first described; the one definite statement he allows himself being "they have no connection with the gout, being found in persons who never had it." Naturally it is on the therapeutic aspect of his subject that Dr. London writes with special fulness, and he is explicit in urging that "suitable dieting has achieved greater results than any measures in use up to the present which aim at bringing about a solution of uric acid." Colchicum, he argues, possesses the power of restoring to the tissues their normal capacity for decomposing uric acid, and he finds no effective substitute for it. Salicylates he regards as valuable for the relief of pain in acute attacks, and he mentions aspirin as particularly serviceable in this respect. The value of alkalies he attributes, in part at least, to their power to produce a direct increase in nitrogenous interchange. Space prevents us making reference to further points of interest, but it is generally satisfactory to find that the broad lines of treatment as at present practised are approved by one who has had unique opportunities for forming a sound judgment. It is not possible to rival the elaborate organisation for the treatment of gout which exists at Carlsbad and which is described in detail in Dr. London's paper. But the principles which he presents and their application in individual instances have no geographical limit, and his essay is therefore of general interest and value. If its main features confirm current methods of practice there are yet many points of detail on which his suggestions may be consulted with great advantage. Fortunately in relation to gout, as also in regard to other diseases, the therapeutic art is not compelled to await the settlement of doctrinal disputes.

